# Resolution of ribosomal stalling by EF-P and ABCF ATPases YfmR and YkpA/YbiT

**DOI:** 10.1093/nar/gkae556

**Published:** 2024-06-29

**Authors:** Hiraku Takada, Keigo Fujiwara, Gemma C Atkinson, Shinobu Chiba, Vasili Hauryliuk

**Affiliations:** Faculty of Life Sciences and Institute for Protein Dynamics, Kyoto Sangyo University, Kamigamo, Motoyama, Kita-ku, Kyoto 603-8555, Japan; Department of Biotechnology, Toyama Prefectural University, 5180 Kurokawa, Imizu, Toyama 939-0398, Japan; Department of Experimental Medical Science, Lund University, 221 00 Lund, Sweden; Faculty of Life Sciences and Institute for Protein Dynamics, Kyoto Sangyo University, Kamigamo, Motoyama, Kita-ku, Kyoto 603-8555, Japan; Department of Experimental Medical Science, Lund University, 221 00 Lund, Sweden; Virus Centre, Lund University, Lund, Sweden; Faculty of Life Sciences and Institute for Protein Dynamics, Kyoto Sangyo University, Kamigamo, Motoyama, Kita-ku, Kyoto 603-8555, Japan; Department of Experimental Medical Science, Lund University, 221 00 Lund, Sweden; Virus Centre, Lund University, Lund, Sweden; University of Tartu, Institute of Technology, 50411 Tartu, Estonia; Science for Life Laboratory, Lund, Sweden

## Abstract

Efficiency of protein synthesis on the ribosome is strongly affected by the amino acid composition of the assembled amino acid chain. Challenging sequences include proline-rich motifs as well as highly positively and negatively charged amino acid stretches. Members of the F subfamily of ABC ATPases (ABCFs) have been long hypothesised to promote translation of such problematic motifs. In this study we have applied genetics and reporter-based assays to characterise the four housekeeping ABCF ATPases of *Bacillus subtilis*: YdiF, YfmM, YfmR/Uup and YkpA/YbiT. We show that YfmR cooperates with the translation factor EF-P that promotes translation of Pro-rich motifs. Simultaneous loss of both YfmR and EF-P results in a dramatic growth defect. Surprisingly, this growth defect can be largely suppressed though overexpression of an EF-P variant lacking the otherwise crucial 5-amino-pentanolylated residue K32. Using *in vivo* reporter assays, we show that overexpression of YfmR can alleviate ribosomal stalling on Asp-Pro motifs. Finally, we demonstrate that YkpA/YbiT promotes translation of positively and negatively charged motifs but is inactive in resolving ribosomal stalls on proline-rich stretches. Collectively, our results provide insights into the function of ABCF translation factors in modulating protein synthesis in *B. subtilis*.

## Graphical Abstract

**Graphical Abstract:**
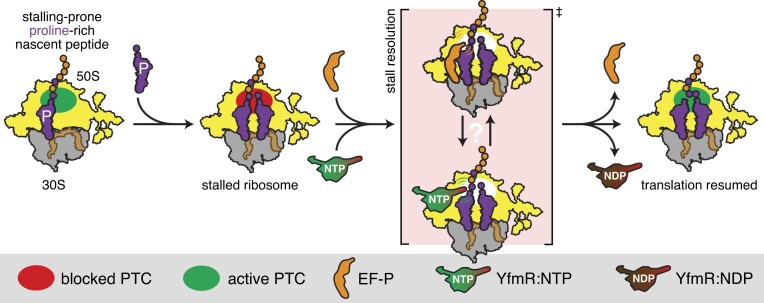

## Introduction

Protein synthesis on the ribosome is assisted by an array of dedicated protein factors that participate in all steps of translation: initiation, elongation, termination and recycling. The most well-studied group of ribosome-associated factors is translational GTPases (1–3). These factors promote the ‘core’ activities of the ribosome: bacterial initiation factor 2, IF2, promotes correct positioning of the initiator fMet-tRNA_i_, elongation factors EF-Tu and EF-G assist the delivery of aminoacyl-tRNA and catalyse ribosomal translocation, respectively, and, acting together with Ribosome Recycling Factor, RRF, EF-G splits the ribosome into subunits after the polypeptide is completed.

While translational GTPases all bind to the ribosomal A (acceptor) site, multiple ‘accessory’ factors act in the E (exit) site. Bacterial elongation factor P, EF-P, accesses the ribosomal peptidyl transferase center, PTC, to relieve ribosomal stalling on proline-rich motifs (4–8). While the C-terminal OB domain of the factor interacts with the mRNA in the E site (4) with the guanosine residue in the first position acting as a recognition element (9), the N-terminal KOW domain stabilises the P-site tRNA in the PTC (4,10). With the notable exception of a group of Actinobacterial species (11,12), in the vast majority of bacteria, PTC stimulation by EF-P requires the posttranslational modification of a conserved lysine residue located in the loop region between beta-strands three and four (β3Ωβ4) of the KOW domain, with specific modifications differing in different bacterial lineages (13–17). In *Bacillus subtilis*, EF-P is modified with a 5-aminopentanol moiety at Lys32 (15) via a multistep assembly pathway that relies on several enzymes: GsaB, YnbB, YmfI, YaaO, YfkA and YwlG (18). GsaB, YnbB and YmfI directly catalyse the EF-P modification while YaaO, YfkA and YwlG are believed to play an indirect role though supporting synthesis of the substrate (18). EF-P is not essential in *B. subtilis*, nor in *Escherichia coli* (18,19). EF-P loss results in a pleiotropic phenotype, which in *B. subtilis* involves compromised sporulation (due to the reduced expression of the Spo0A transcription factor) (20) and swarming mobility (due to the reduced expression of multiple swarming mobility-associated proteins, including FliP and FlhP) (15). However, EF-P is essential in other bacterial species such as *Neisseria meningitidis* (21), and the eukaryotic EF-P orthologue, eIF5A, is essential in yeast (22) and flies (23). Notably, in addition to its role in promoting translation elongation on proline-rich stretches, eIF5A also plays a crucial role in translation termination (24); no similar function has been shown for EF-P. Finally, numerous bacterial species, including *E. coli*, encode a second EF-P paralog, named EF-P like (EfpL) or elongation factor P-like protein (YeiP) (9). While the two paralogues have overlapping functions, EfpL and EF-P display a certain degree of functional diversification as they alleviate ribosomal stalling on distinct proline-containing motifs (9).

**Figure 1.**
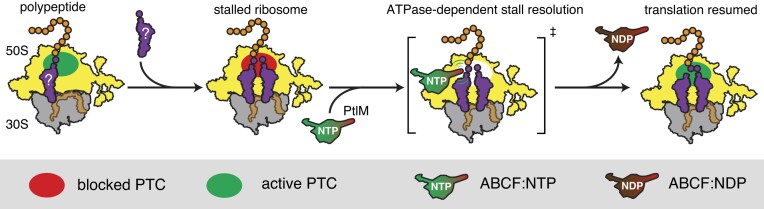
Generalised model for ribosomal rescue by housekeeping ABCF ATPases. Translation though ‘challenging’ amino acid motifs stalls the ribosome, inactivating the PTC. Housekeeping ABCF binds to the ribosomal E site and accesses the PTC with its P-site tRNA interaction motif (PtIM) domain. Following the NTPase-dependent reset of the PTC by the factor, the ABCF departs from the E site; translation resumes. The exact nature of the stalls resolved by the individual ABCFs is currently unclear.

The F subfamily of ABC ATPases (ABCFs) comprises another group of E-site-binding translation factors in bacteria (25,26). The family includes both antibiotic resistance (ARE) factors as well as housekeeping proteins that assist protein synthesis and ribosome assembly (27–31). The *B. subtilis* genome encodes five ABCFs: a dedicated antibiotic resistance factor VmlR (32) and housekeeping factors YdiF, YfmM, YfmR/Uup and YkpA/YbiT (29). The exact functions of housekeeping ABCFs are unclear. Multiple lines of evidence suggest that, analogous to how ARE ABCF resolve ribosome stalling caused by antibiotics (33–37), housekeeping ABCFs resolve other stalling events in an NTPase-dependent manner by reaching into the PTC with their P-site tRNA interaction motif (PtIM) domain (27,28,38) (Figure 1). *E. coli* EttA is by far the best characterised housekeeping ABCF, with structural and biochemical evidence indicating a role in the regulation of the first rounds of translation elongation (27,28,38). The EttA subfamily evolved from the diversity of the Uup ABCF subfamily (29). Several studies suggest a non-ribosomal role for Uup in resolving DNA repair intermediates (39,40) and transposon excision (41). At the same time, disruption of the *uup* gene in *E. coli* strain lacking an accessory translational GTPase BipA moderately exacerbates the cold sensitivity and ribosome assembly defects of the Δ*bipA* strain, while Uup overexpression of suppresses the defects (33). Given the BipA’s chaperone-like role in late stages of the 50S assembly (42), this genetic interaction is suggestive of Uup playing a role in translation or/and ribosome assembly. The ribosomal function of Uup is further supported by specific inhibition of protein synthesis upon expression of the ATPase-deficient (EQ_2_) variant due to non-productive association of the Uup-EQ_2_ variant with the ribosome (33,43). Ectopically overexpressed ABCF-EQ_2_ variants preferentially bind to the vacant E site of 70S initiation complexes (IC) (27,28) which has been successfully exploited for immunoaffinity-based purification of ABCF-EQ_2_:IC complexes for structural studies (36,37,44,45).

We have characterised the potential functional overlap between the two classes of E-site-inspecting factors in *B. subtilis*: EF-P and housekeeping ABCFs. While the two classes cannot operate on the ribosome simultaneously due to a steric clash, we show a genetic between *epf* and *yfmR*, with functional assays demonstrating that *B. subtilis* YfmR is able to resolve ribosome stalling on Asp-Pro motifs in the absence of EF-P. Furthermore, we demonstrate that YkpA/YbiT promotes translation of EF-P-insensitive positively and negatively charged motifs.

## Materials and methods

### Phylogenetic analysis

Representative sequences were selected from Murina *et al.* (29). Sequences were aligned with MAFFT v6.861b with the L-INS-i strategy (46) and visualised in AliView (47). Positions with 50% gaps were removed with trimAl v. 1.4.rev6 (48). Maximum likelihood phylogenetic analysis was carried out with IQ-Tree v. 2.2.2.6 (49) at the IQ-Tree web server (50) with the best fit model selected by the program (Q.pfam + I + G4), SH-aLRT and ultrafast bootstrap and branch support testing (1000 replicates) (51).

### Construction of bacterial strains and plasmids

The strains, plasmids and oligonucleotides used in this study as well as description of strain construction are provided in Supplementary Table S1. Plasmids were constructed by standard cloning methods: PCR, PrimeSTAR mutagenesis (Takara), and Gibson assembly. Marker-less gene deletion mutants of *efp* (BCHT209), *ydiF* (BCHT212), *yfmM* (BCHT213), *yfmR* (BCHT214), *ykpA* (BCHT215), *gsaB* (BCHT332), *yaaO* (BCHT333), *yfkA* (BCHT334), *ymfI* (BCHT335) and *ynbB* (BCHT336) were constructed by excising the antibiotic resistance cassette by the Cre-loxP system as described previously (52). Briefly, *B. subtilis* strains were transformed with pMK2, a pLOSS*-based Ts plasmid harbouring *cre*. To select for the excision of the resistance marker flanked by *loxP*, the resulting strains were grown overnight at 37°C on LB agar medium supplemented with 1 mM isopropyl β-D-1-thiogalactopyranoside (IPTG) and 100 μg/ml spectinomycin. Finally, to promote the loss of the pMK2 plasmid, the strains were grown overnight at 37°C on LB agar medium without spectinomycin. The loss of pMK2 was confirmed by the absence of spectinomycin resistance.

### Sucrose gradient fractionation and immunoblotting

The experiments were performed as described previously (53). Briefly, *B. subtilis* strains were grown at 37°C in 40 ml LB cultures until the OD_600_ of 0.8, cells collected by centrifugation and dissolved in 0.5 ml of HEPES:Polymix buffer [5 mM Mg(OAc)_2_] (53), lysed by FastPrep homogenizer (MP Biomedicals) and the resultant lysates clarified by centrifugation. 10 *A*_260_ units of each extract were loaded onto 10–35% (w/v) sucrose density gradients prepared in HEPES:Polymix buffer [5 mM Mg(OAc)_2_] and the gradients were resolved by ultracentrifugation at 36 000 rpm for 3 h at 4°C. Both preparation and fractionation of gradients was done using Biocomp Gradient Station (BioComp Instruments); *A*_260_ was used as a readout during the fractionation.

### Ribosome stalling reporter assay

Ribosome stalling reporters were based on the reporters developed by Chadani and colleagues (54). To construct the reporters, the test motif-encoding DNA segments encoding pairs of either homodecapeptides (A_10_, K_10_, R_10_, E_10_ or D_10_) or (DP)_5_ hetrodecapeptides connected via a (GS)_2_ liker were intercalated into the GFP-SUMO-coding ORF between the segments encoding the two domains. The construction was achieved via one-step PCR amplification with partially complementary ssDNA oligonucleotides. The use of a unified (GS)_2_ liker simplified the construction of the reporters. The presence of a relatively bulky C-terminal SUMO tag allowed for efficient SDS PAGE separation of the full-length product from the truncated product generated upon ribosomal stalling on the test motif.

Reporters were expressed under the control of P*_hy-spank_* IPTG-inducible promoter (55) from a self-replicated pHT01-based plasmid carrying a kanamycin resistance marker (56). ABCF- and EF-P-coding genes were cloned on the pSHP2 plasmid (provided by Dr Henrik Strahl von Schulten) under the control of P*_xy_* xylose-inducible promoter and integrated into the *amyE* locus. Individual reporter plasmids were amplified by EquiPhi29 polymerase (Thermo Fisher Scientific) and transformed into recipient *B. subtilis* strains. The resulting strains were grown overnight at 37°C on LB plates supplemented with 3 μg/ml kanamycin. After isolating single colonies twice on LB plates supplemented with 3 μg/ml kanamycin, fresh colonies of *B. subtilis* harbouring reporter plasmids were used to inoculate 1-ml LB medium cultures dispensed into plastic 96 deep-well plates (Treff Lab). The cultures were grown at 30°C for 18 h with shaking at 1200 rev per min using DWMax M·BR‐034P constant temperature incubator shaker (Taitec). 20 μl of individual overnight cultures were then used to inoculate 1 ml cultures (LB supplemented with 3 μg/ml kanamycin as well as inducers: 1 mM IPTG and 0.3% xylose) dispensed into plastic 96 deep-well plates. 1-ml experimental cultures were grown at 37°C with shaking until OD_600_ of 1.0, 0.75  ml aliquots collected, combined with 83 μl of 50% TCA and kept on ice for 5 min. After centrifugation at 13 500 rpm for 2 min at 4°C, cell pellets were resuspended in 500 μl of 0.1 M Tris-HCl (pH 6.8). After one more round of 2-min centrifugation at 13 500 rpm at 4°C, cell pellets were resuspended in 50 μl of lysis buffer (0.5 M sucrose, 20 mM MgCl_2_, 1 mg/ml lysozyme, 20 mM HEPES:NaOH, pH 7.5) and incubated at 37  °C for 10 min. Next, an equal volume of 2 × SDS sample buffer (4% SDS, 30% glycerol, 250 mM Tris pH 6.8, 1 mM DTT, saturated bromophenol blue) was added, and the lysates were denatured at 85°C for 5 min. Proteins were resolved on 11% SDS-PAGE and transferred to a PVDF membrane. GFP-tagged proteins were detected using anti-GFP (Wako, mFX75, 1:5  000 dilution) antibodies combined with Goat Anti-Mouse IgG (H + L) HRP Conjugate (Bio-Rad). Images were acquired using Amersham Imager 600 (GE Healthcare) luminoimager and analysed in ImageJ (57). The stalled fraction was quantified by dividing the stalled (short) product signal by the total signal (short and full-length combined). All experiments were performed as three biological replicates; quantification is shown as mean ± standard deviation.

## Results

### *B. subtilis* YfmR is a member of the Uup/EttA ABCF clade

**Figure 2.**
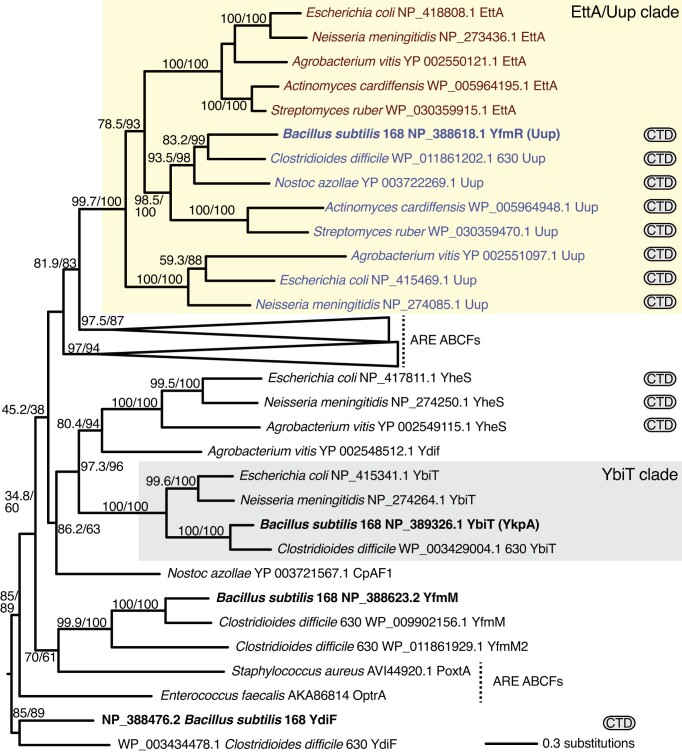
*B. subtilis* YfmR is an Uup ABCF subfamily member within the EttA/Uup clade. Maximum likelihood phylogeny of ABCF protein representatives. *B. subtilis* housekeeping ABCFs YfmR, YdiF, YfmM and YkpA (YbiT) are shown in bold. The CTD logo indicates the presence of a C-terminal domain. Numbers in parentheses are SH-aLRT support (%)/ultrafast bootstrap support (%). Only branches with >60% bootstrap support are labelled. Branch length is proportional to the number of substitutions as per the lower right key.

YfmR is classified with ABCF Hidden Markov models as a member of the Uup subfamily (29). The Uup subfamily is not monophyletic, but rather is paraphyletic to the EttA subfamily, which arose from an Uup-like progenitor (Figure 2, (29)). Uup subfamily members typically carry a C-terminal domain (CTD), which is absent in EttA, suggesting this domain was lost after the *uup* gene duplication that gave rise to EttA. The monophyly of the Uup + EttA clade is strongly supported (99.7% SH-aLRT and 100% bootstrap support).

### Simultaneous disruption of *yfmR* and *efp* results in a synthetic growth defect

**Figure 3.**
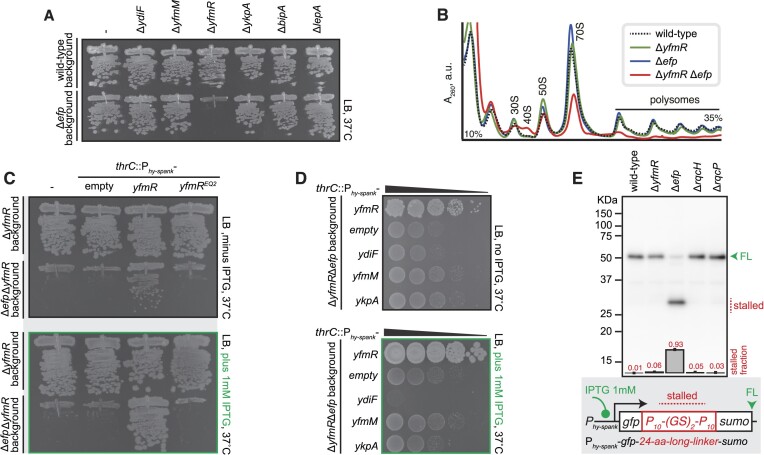
Simultaneous loss of *yfmR* and *efp* results in a dramatic growth defect. (**A**) Effects of mutations targeting housekeeping ABCFs and non-essential translational GTPases BipA and LepA on the growth of wild-type and Δ*efp B. subtilis*. The Δ*ydiF* (strains BCHT174 and BCHT195; wt and Δ*efp* background, respectively), Δ*yfmM* (strains BCHT171 and BCHT192), Δ*yfmR* (strains BCHT170 and BCHT191), Δ*ykpA* (strains BCHT170 and BCHT191), Δ*bipA* (strains BCHT172 and BCHT193) and Δ*lepA* (strains BCHT173 and BCHT194) *B. subtilis* were grown on solid LB medium at 37°C. Wild-type *B. subtilis* and the isogenic Δ*efp* mutant (strain BCHT175) were streaked as controls. (**B**) Overexpression of the ATPase-deficient YfmR-EQ_2_ mutant does not rescue the growth defect of Δ*yfmR*Δ*efp B. subtilis*. *B. subtilis* strains expressing wild-type YfmR (BCHT385 and BCHT389; Δ*yfmR* or Δ*yfmR*Δ*efp* backgrounds, respectively) or YfmR-EQ_2_ (strains BCHT386 and BCHT390) were grown on solid LB medium with (*lower panel*) or without (*upper panel*) 1 mM IPTG. Δ*yfmR* mutant (strains BCHT181 and BCHT170) and Δ*efp*Δ*yfmR* mutant (strains BCHT187 and BCHT191), both with or without integration of the empty vector were streaked as controls. (**C**) Effects of housekeeping ABCF overexpression on the growth of Δ*efp*Δ*yfmR B. subtilis*. YfmR (strain BCHT385 and BCHT389), YdiF (strain BCHT440 and BCHT435), YkpA (strain BCHT441 and BCHT436) and YfmM (strain BCHT442 and BCHT437) were overexpressed in either Δ*yfmR* or Δ*yfmR*Δ*efp B. subtilis* growing on solid LB medium with (*lower panel*) or without (*upper panel*) 1 mM IPTG. (**D**) Polyproline stalling reporter (GFP-P_10_-(GS)_2_-P_10_-SUMO, pCHT55) detects ribosomal stalling in Δ*efp* but not Δ*yfmR B. subtilis*. The reporter was expressed in wild-type, Δ*yfmR* (strain BCHT214), Δ*efp* (strain BCHT214), Δ*rqcH* (strain BCHT58) or Δ*rqcP* (strain BCHT56) *B. subtilis* and detected with anti-GFP antibodies. The full-length product is indicated with a green arrowhead and the stalled product is indicated with a red dotted line. Fraction of the stalled (short) product was quantified from three independent biological replicates and shown as mean ± standard deviation.

We took a genetic approach to probe the functional interactions between housekeeping ABCFs and EF-P in the *B. subtilis* 168 strain. While genomic disruptions of individual ABCF genes in the wild-type background do not affect *B. subtilis* growth on LB medium at the optimal temperature (37°C), deletion of *yfmR*—but not any of the other three housekeeping ABCFs—results in severe growth defect in the Δ*efp* background (Figure 3A). Sucrose gradient centrifugation experiments reveal the low abundance of polysomes as well as accumulation of 40S ribosome assembly intermediates in the Δ*efp* Δ*yfmR* double deletion strain, consistent with perturbed protein synthesis (Supplementary Figure S1). Finally, no genetic interaction was observed for *efp* and poorly understood translational GTPases *bipA* and *lepA* (Figure 3A), suggesting, expectedly, that these factors are not operating together with EF-P.

We have complemented the double deletion Δ*efp* Δ*yfmR* strain with either wild-type or the ATPase-deficient EQ_2_ variant of YfmR expressed under the control of IPTG-inducible P*_hy-spank_* promoter (55). Even in the absence of IPTG, leaky expression of the wild-type protein partially suppressed the growth defect; addition of 1 mM IPTG resulted in full suppression (Figure 3B). Low-level expression of the ATPase-deficient YfmR-EQ_2_ driven by the native Shine-Dalgarno motif fails to complement, consistent with the ATPase activity being essential (Figure 3B). Next, we tested whether ectopic overexpression of housekeeping ABCFs could suppress the growth defect of the Δ*efp* Δ*yfmR* strain. While P*_hy-spank_*-driven overexpression of YfmM and YkpA/YbiT has no effect, overexpression of YdiF further exacerbates the growth defect of Δ*efp* Δ*yfmR B. subtilis* (Figure 3C). A genetic interaction between *efp* and *ydiF* has previously been shown earlier by Hummels and Kearn who have demonstrated that the swarming defect of the Δ*efp B. subtilis* strain can be suppressed by loss-of-function mutations in *ydiF* (58). Notably, in the absence of IPTG, low-level leaky expression of YfmM and YkpA partially suppresses the growth defect of the Δ*efp* Δ*yfmR* strain, which could suggest partial functional redundance between YfmR and these two ABCF ATPases (see the section *Redundancy and specialisation of* B. subtilis *ABCFs*, below).

### YfmR is not essential for efficient translation of polyproline stretches in *efp*^+^ *B. subtilis*

There can be several alternative explanations for the strong genetic interaction between *efp* and *yfmR*. One possibility is that EF-P and YfmR are both required for translation of proline-rich motifs, providing partially redundant solutions to this stalling problem. To test this hypothesis we used a homopolymeric polyproline stalling reporter based on that developed by Chadani and colleagues (54). The reporter gene encodes an N-terminal GFP and C-terminal SUMO tag linked by a 24-amino-acid-long linker with a sequence of P_10_-(GS)_2_-P_10_; two 10-amino-acid-long stalling motifs connected by a flexible ‘joint’ that is not stalling-prone. The gene encoding the reporter was cloned on a self-replicated plasmid pHT01-based plasmid (56) and the expression was driven by IPTG-inducible P*_hy-spank_* promoter. While the full-length version of the reporter construct was efficiently produced in the wild-type *B. subtilis*, only a fraction of the full-length product is synthesised in the Δ*efp* strain, with the majority of the ribosomes stalling and generating a short version of the reporter (Figure 3D). The Δ*yfmR* strain behaved like the wild-type, with no short versions of the reporter being produced. Therefore, we concluded that YfmR is unlikely to be involved in translation of strongly stalling polyproline stretches and that EF-P and YfmR synergise in other, yet-undefined stalling motifs.

### EF-P variants lacking the K32 residue or its 5-aminopentanol modification can still efficiently suppress of the growth defect of Δ*efp* Δ*yfmR B. subtilis*

**Figure 4.**
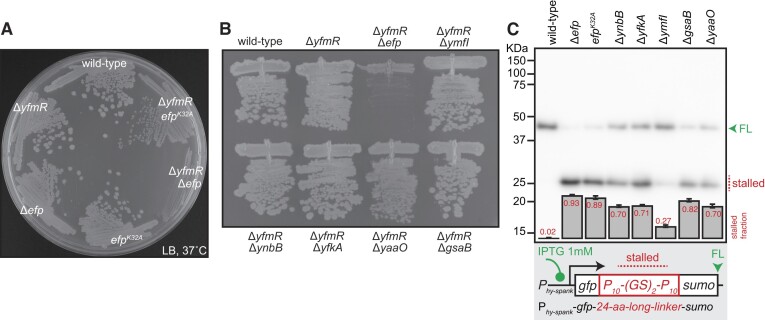
5-amino-pentanolylated residue K32 of EF-P is not essential for supporting a near-wild-type fitness of the Δ*yfmRB. subtilis*. (**A**) Effect of the *efp^K32A^*mutation on the negative genetic interaction between *yfmR* and *efp*. Wild-type, Δ*yfmR* (strain BCHT170), Δ*efp* (strain BCHT175), *efp^K32A^* (strain BCHT8765), Δ*yfmR efp^K32A^* (strain BCHT879) and Δ*yfmR*Δ*efp* (strain BCHT191) *B. subtilis* were grown on solid LB medium at 37°C. (**B**) Effects of disruption of the genes involved in the 5-aminopentanol modification of the K32 residue of EF-P on the growth of Δ*yfmR B. subtilis*. Wild-type, Δ*yfmR* (strain BCHT170), Δ*yfmR*Δ*efp* (strain BCHT191), Δ*yfmR*Δ*ymfI* (strain BCHT396), Δ*yfmR*Δ*ynbB* (strain BCHT395), Δ*yfmR*Δ*yfkA* (strain BCHT394), Δ*yfmR*Δ*yaaO* (strain BCHT393) and Δ*yfmR*Δ*gsaB* (strain BCHT392) *B. subtilis* were grown on solid LB medium at 37°C. (**C**) Effects of the K32A substitution and genetic disruption of the K32 5-aminopentanol modification on the ribosomal stalling on polyproline. GFP-P_10_-(GS)_2_-P_10_-SUMO reporter (pCHT55) was expressed in wild-type, Δ*efp* (strain BCHT214), *efp^K32A^* (strain BCHT765), Δ*ynbB* (strain BCHT336), Δ*yfkA* (strain BCHT334), Δ*ymfI* (strain BCHT335), Δ*gsaB* (strain BCHT332) or Δ*yaaO* (strain BCHT333) *B. subtilis* and detected with anti-GFP antibodies. The full-length product is indicated with a green arrowhead and the stalled product is indicated with a red dotted line. Fraction of the stalled (short) product was quantified from three independent biological replicates and shown as mean ± standard deviation.

With the notable exception of Actinobacterial EF-P (11), the posttranslational modification of a conserved lysine residue is crucial for the factor's functionality in resolving polyproline stalling, both in living cells and a reconstituted biochemical system (6,8,14,18). However, it is conceivable that the modification is not essential for the hypothetical activity on which EF-P and YfmR work together. To probe this hypothesis, we tested whether EF-P lacking the modification of conserved K32 residue—or the K32 residue altogether—can suppress the synthetic growth defect of Δ*efp* Δ*yfmR B. subtilis*. Surprisingly, a Δ*yfmR B. subtilis* strain expressing the K32A EF-P variant does not phenocopy the severe growth defect of the Δ*efp* Δ*yfmR* double knockout (Figure 4A). Next, we tested the genetic interaction between *yfmR* and the genes involved in the 5-aminopentanol modification of the K32 residue: *yaaO*, *yfkA*, *ynbB*, *gsaB* and *yfmI* (18). Previous mass spectrometry studies have established that while in Δ*yaaO* and Δ*yfkA B. subtilis* strains EF-P retains low levels of 5-aminopentanol modification, in Δ*ynbB* and Δ*gsaB* strains the K32 residue is acetylated and in Δ*ymfI* it carries 5-aminopentanone instead of 5-aminopentanol (18,59). None of the tested genes strongly genetically interact with the *yfmR* disruption: none of the double-knockout strains display a severe growth defect either; a minor growth defect is detectable in Δ*yaaO* Δ*yfmR* (Figure 4B).

We next used our polyproline stalling reporter [GFP-P_10_-(GS)_2_-P_10_-SUMO] to assess the effects of the K32A substitution—as well as disruption of the 5-aminopentanol modification pathway—on EF-P’s functionality in promoting translation elongation on polyproline stretches. The K32A substitution phenocopied the Δ*efp* strain (Figure 4C). This result is in good agreement with analogous *in vivo* assays by Rajkovic and colleagues who tested an array of different PPX stalling peptides such as PPW, PPG, PPP, PPR (15). Mutations in the enzymes implicated in the 5-aminopentanol modification of the K32 residue strongly—although not as completely as the K32A substitution—compromised EF-P’s activity (Figure 4C). The weakest effect was observed in the case of disruption of YfmI, an enzyme which catalyses the last step in EF-P modification, the reduction EF-P-5-aminopentanone to EF-P-5-aminopentanol (59).

### Overexpression of YfmR/Uup alleviates the ribosomal stalling on Asp-Pro motifs in Δ*efp B. subtilis*

We next tested whether overexpression of YfmR can improve the ability of the *B. subtilis* translational apparatus to synthesise challenging motifs. Inspired by the work by Chadani and colleagues (54), we used a series of diverse GFP-*linker*-SUMO reporters with different linker sequences. The presence of a C-terminal SUMO domain allowed efficient separation of the stalled product that lacks this domain from the full length version. Specifically, we tested homopolymeric P_10_-(GS)_2_-P_10_ and D_10_-(GS)_2_-D_10_ as well as ‘mixed’ (DP)_5_-(GS)_2_-(DP)_2_. The two proline-rich linkers are expected to specifically cause ribosomal stalling in Δ*efp B. subtilis* while the highly negatively charged poly-Asp motif is in general challenging for translation (54). Finally, the A_10_-(GS)_2_-A_10_ motif was used as a negative control as no stalling is expected in wild-type and Δ*efp B. subtilis*. The FLAG-tagged versions of either wild-type or functionally compromised proteins (ATPase-deficient EQ_2_ variants) were expressed under the control of a xylose-inducible P*_xy_* promotor. In this case YfmR was expressed under the control of strong Shine-Dalgarno motif, and this strong expression of YfmR-EQ_2_ is associated with a growth defect, most likely due to non-productive ribosomal association inhibiting translation. We have observed analogous effects in the case of ATPase-deficient housekeeping ABCFs in *E. coli* (29). Overexpression of either wild-type or K32A-substituted EF-P was used as two additional controls, and the reporter assays were performed either in wild-type or a Δ*efp* genetic background. Anti-FLAG immunoblotting experiments revealed that wild-type and K32A-substituted EF-P variants are expressed at similar levels, while YfmR-EQ_2_ is expressed at a lower level than the wild-type factor, consistent with the inhibitory function of the ATPase-deficient ABCF factor on protein synthesis (Supplementary Figure S1).

**Figure 5.**
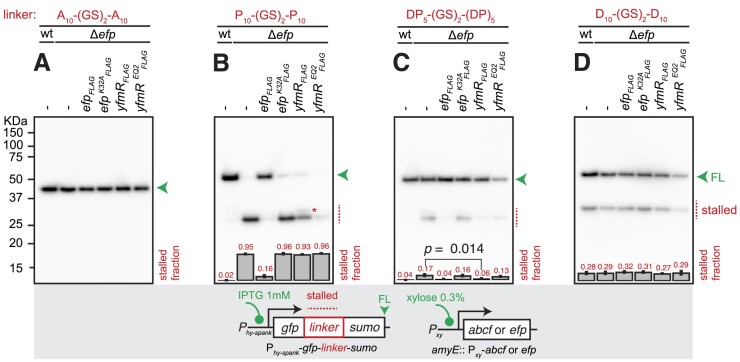
Overexpression of YfmR/Uup ABCF alleviates the ribosomal stalling on Asp-Pro motifs. Effects of EF-P and YfmR overexpression on ribosomal stalling on polyproline, polyaspartic acid as well as mixed Asp-Pro stalling motifs. GFP-A_10_-(GS)_2_-A_10_-SUMO (pCHT54) (**A**), GFP-P_10_-(GS)_2_-P_10_-SUMO (pCHT55) (**B**), GFP-(DP)_5_-(GS)_2_-(DP)_5_-SUMO (pCHT12) (**C**) and GFP-D_10_-(GS)_2_-D_10_-SUMO (pCHT15) (**D**) reporters were expressed in wild-type, Δ*efp* (strain BCHT214) as well as in Δ*efp B. subtilis* expressing either *efp_FLAG_*(strain BCHT1367), *efp^K32A^_FLAG_*(strain BCHT1368), *yfmR_FLAG_* (strain BCHT1369) or *yfmR^EQ2^_FLAG_* (strain BCHT1370) under the control of xylose promoter. All reporters were detected with anti-GFP antibodies. The full-length product is indicated with a green arrowhead and the stalled product is indicated with a red dotted line. A red asterisk indicates a larger stalled reporter product observed upon overexpression of YfmR-FLAG. Fraction of the stalled (short) product was quantified from three independent biological replicates and shown as mean ± standard deviation. An unpaired one-tailed Student's *t*-test was used to compare Δ*efp* and Δ*efp* + *efp_FLAG_* groups on the panel (**C**). The effect size, measured as the ratio of sample means is 3.14 and the p-value is 0.014. Anti-FLAG immunoblotting for detection of wild-type and EQ_2_ YfmR as well as wild-type and K32A EF-P is shown on the Supplementary Figure S1. The three individual experimental replicates of the panel (C) are shown on the Supplementary Figure S2.

As expected, no truncated versions of the control GFP-A_10_-(GS)_2_-A_10_-SUMO reporter are detectable by anti-GFP antibodies regardless of the strain background and the protein expressed (Figure 5A). Near 100%-efficient stalling on P_10_-(GS)_2_-P_10_ in Δ*efp B. subtilis* is fully resolved upon overexpression of wild-type EF-P; overexpression of the K32A-substituted variant failed to resolve the stall (Figure 5B). While overexpression of wild-type YfmR did not restore the production of the full-length reporter, it resulted in formation of a longer stalled product (marked with a red asterisk on Figure 5B), indicative of a possible modest stimulatory effect. Expression of YfmR-EQ_2_ decreased both the full-length and stalled reporter signal, most likely due to translation inhibition caused by the factor being locked in the ribosomal E-site. Experiments with a weaker EF-P-sensitive staller, (DP)_5_-(GS)_2_-(DP)_2_, indicated the ability of YfmR to resolve ribosomal stalls on proline-rich motifs: expression of either EF-P or YfmR abrogated the stalled signal, and the effect was specific for wild-type factors (Figure 5C, Supplementary Figure S2). Finally, we tested whether overexpression of either EF-P or YfmR could overcome ribosomal stalling on acidic poly-Asp motifs (Figure 5D). In agreement with EF-P not being able to resolve the poly-Asp stalling, the strength of stalled signal was similar in wild-type and Δ*efp B. subtilis*. Overexpression of neither of the factors could resolve ribosomal stalling on the D_10_-(GS)_2_-D_10_ motif.

### *B. subtilis* YkpA/YbiT promotes translation of positively and negatively charged motifs

Prompted by our results with YfmR, we decided to examine the possible involvement of all of the four *B. subtilis* ABCFs—YfmR, YdiF, YfmM and YkpA/YbiT—in translating diverse challenging sequences. As stretches of both highly positively and negatively charged amino acids can be challenging for the ribosome (54,60–63), we have also included polybasic [K_10_-(GS)_2_-K_10_ and R_10_-(GS)_2_-R_10_] motifs as well as an additional polyacidic [D_10_-(GS)_2_-D_10_] linker, respectively.

**Figure 6.**
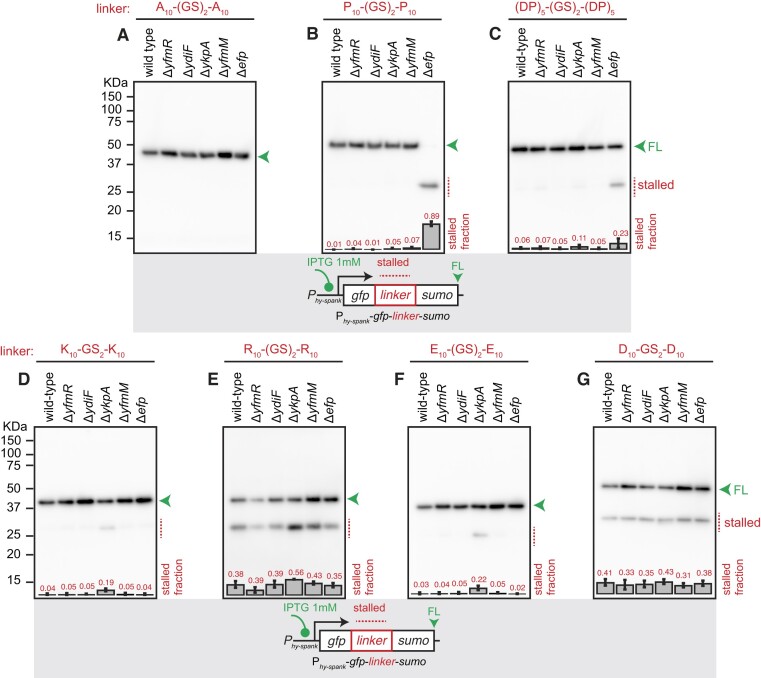
YkpA/YbiT loss results in mild ribosomal stalling on positively charged polylysine and negatively charged polyglutamic acid motifs. Effects of ABCF and EF-P gene disruption on ribosomal stalling on polyproline, Asp-Pro as well as negatively and positively charged homopolymeric motifs. GFP-A_10_-(GS)_2_-A_10_-SUMO (pCHT54) (**A**), GFP-P_10_-(GS)_2_-P_10_-SUMO (pCHT55) (**B**), GFP-(DP)_5_-(GS)_2_-(DP)_5_-SUMO (pCHT12) (**C**), GFP-K_10_-(GS)_2_-K_10_-SUMO (pCHT13) (**D**), GFP-R_10_-(GS)_2_-R_10_-SUMO (pCHT56) (**E**), GFP-E_10_-(GS)_2_-E_10_-SUMO (pCHT11) (**F**) and GFP-D_10_-(GS)_2_-D_10_-SUMO (pCHT15) (**G**) reporters were expressed in wild-type, Δ*yfmR* (strain BCHT214), Δ*ydiF* (strain BCHT212), Δ*ykpA* (strain BCHT215) and Δ*yfmM* (strain BCHT213). The full-length product is indicated with a green arrowhead and the stalled product is indicated with a red dotted line. All reporters were detected with anti-GFP antibodies. Fraction of the stalled (short) product was quantified from three independent biological replicates and shown as mean ± standard deviation.

We tested all of the reporters listed above in wild-type and Δ*efp B. subtilis* as well as the four Δ*abcf B. subtilis* strains: Δ*yfmR*, Δ*ydiF*, Δ*ykpA* or Δ*yfmM*. As expected, all of the strains produce exclusively the full-length version of the GFP-A_10_-(GS)_2_-A_10_-SUMO reporter (Figure 6A). None of the ABCFs are crucial for translation of polyproline stretches in *efp*^+^*B. subtilis*: while the short, stalled version of the GFP-P_10_-(GS)_2_-P_10_-SUMO reporter is dominant in Δ*efp B. subtilis*, only the full-length signal is detectable in all of the Δ*abcf* strains (Figure 6B). An analogous result was obtained with GFP-(DP)_5_-(GS)_2_-(DP)_2_-SUMO, although the strength of stalling in the Δ*efp* background is considerably weaker: the stalled form constitutes about 20% of the total signal (Figure 6C). Experiments with polybasic stallers yielded non-trivial results. In the case of K_10_-(GS)_2_-K_10_ linker we detected specific (but relatively weak) stalling in the Δ*ykpA* (Δ*ybiT*) strain (Figure 6D). While the R_10_-(GS)_2_-R_10_ motif was challenging for all of the tested strains, the strongest stalling signal was, again, observed in the case of Δ*ykpA B. subtilis*. Furthermore, weak Δ*ykpA*-specific stalling was observed in the case of E_10_-(GS)_2_-E_10_ polyacidic linker (Figure 6F). The polyacidic D_10_-(GS)_2_-D_10_ reporter was equally challenging for all of the tested strains (Figure 6G). Collectively, our results suggest that YbiT could be assisting the ribosome in negotiating charged amino acid patches.

### Redundancy and specialization of *B. subtilis* ABCFs

We wondered whether the reason for the modest stalling effects we observed upon disrupting individual *abcf* genes could be the partial functional redundance between the factors analogous to that demonstrated for paralagous EF-P and EfpL (9). To test this hypothesis, we created a set of *efp^+^ B. subtilis* strains in which we disrupted the ABCF genes in pairwise combinations, in combinations of three and, finally, a Δ*4abcf* strain in which all the four genes are disrupted—in which to test our stalling reporters. The strains displayed no growth defects or increased sensitivity to low concentrations of translation targeting antibiotics c (Supplementary Figure S3)

**Figure 7.**
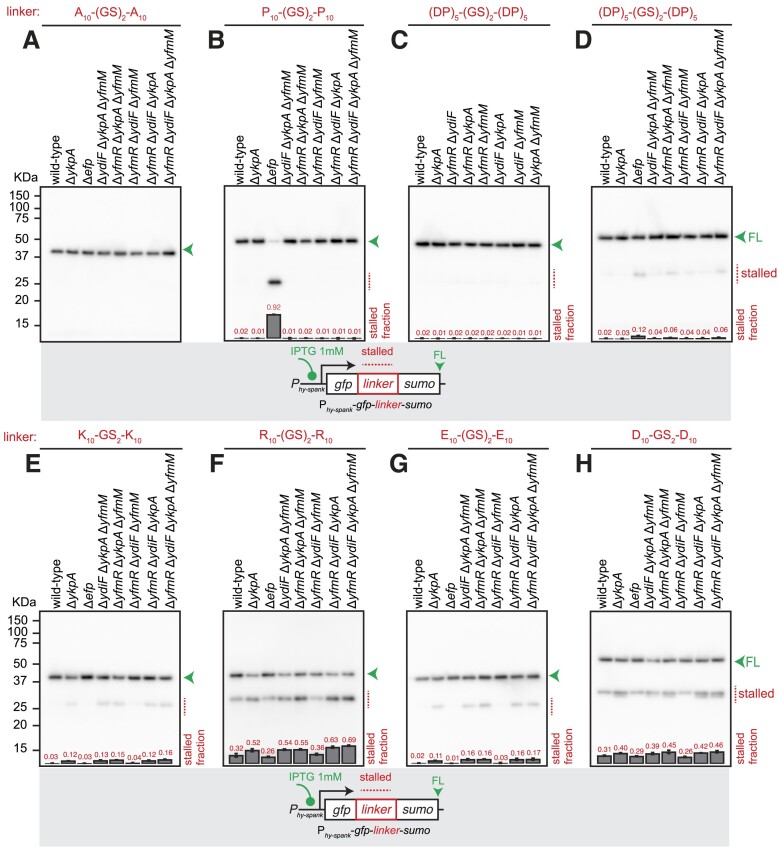
Probing the functional redundancy of *B. subtilis* ABCFs through combinatorial gene disruptions. Effects of combinatorial ABCF gene disruption on ribosomal stalling on polyproline, Asp-Pro as well as negatively and positively charged homopolymeric motifs. GFP-A_10_-(GS)_2_-A_10_-SUMO (pCHT54) (**A**), GFP-P_10_-(GS)_2_-P_10_-SUMO (pCHT55) (**B**), GFP-(DP)_5_-(GS)_2_-(DP)_5_-SUMO (pCHT12) (**C, D**), GFP-K_10_-(GS)_2_-K_10_-SUMO (pCHT13) (**E**), GFP-R_10_-(GS)_2_-R_10_-SUMO (pCHT56) (**F**), GFP-E_10_-(GS)_2_-E_10_-SUMO (pCHT11) (**G**) and GFP-D_10_-(GS)_2_-D_10_-SUMO (pCHT15) (**H**) reporters were expressed in wild-type, Δ*ykpA* (strain BCHT215), Δ*efp* (strain BCHT214), Δ*ydiF*Δ*ykpA*Δ*yfmM* (strain BCHT1388), Δ*yfmR*Δ*ykpA*Δ*yfmM* (strain BCHT1387), Δ*yfmR*Δ*ydiF*Δ*yfmM* (strain BCHT1386), Δ*yfmR*Δ*ydiF*Δ*ykpA* (strain BCHT1385), Δ*yfmR*Δ*ydiF*Δ*ykpA*Δ*yfmM* (the Δ*4abcf* strain BCHT1389). The full-length product is indicated with a green arrowhead and the stalled product is indicated with a red dotted line. All reporters were detected with anti-GFP antibodies. Fraction of the stalled (short) product was quantified from three independent biological replicates and shown as mean ± standard deviation.

As expected, we did not detect any stalling on the A_10_-(GS)_2_-A_10_ motif in any of the strains (Figure 7A, Supplementary Figure S4A). Similarly, no stalling was observed on the P_10_-(GS)_2_-P_10_ motif either, which is expected for *efp^+^ B. subtilis* (Figure 7B, Supplementary Figure S4B). While stalling is not detected on the (DP)_5_-(GS)_2_-(DP)_2_ motif in either of the tested double-KO strains (Figure 7C), modest stalling is detectable in the Δ*yfmR* Δ*ykpA* Δ*yfmM* background (Figure 7D). The effect is not exacerbated by the additional loss of YdiF in the Δ*4abcf* strain (Figure 7D), suggesting that YdiF does not contribute to resolution of the stalls. Taken together with the observation that leaky overexpression of YfmM and YkpA partially suppresses the growth defect of the Δ*efp* Δ*yfmR* strain (Figure 3C), this result further strengthens the idea of a functional overlap between YfmR, YkpA and YfmM. Additional disruptions of ABCF genes in the Δ*ykpA B. subtilis* strain does not exacerbate the stalling on the polybasic K_10_-(GS)_2_-K_10_ and R_10_-(GS)_2_-R_10_ (Figure 7E, F, Supplementary Figure S4C, D) as well as polybasic in E_10_-(GS)_2_-E_10_ and D_10_-(GS)_2_-D_10_ (Figure 7G, H, Supplementary Figure S4E, F). This suggests that YkpA is specifically competent in resolving ribosomal stalls on charged motifs.

### Simultaneous disruption of *bipA* and *efp* results in a cold sensitivity in *B. subtilis*

**Figure 8.**
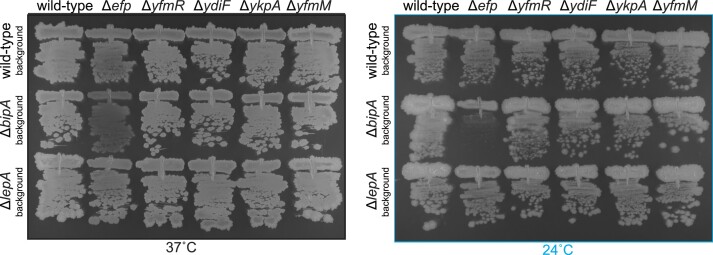
Simultaneous loss of *bipA* and *efp* results in a severe cold sensitivity. Effects of mutations targeting housekeeping ABCFs and EF-P the growth of wild-type, Δ*bipA* and Δ*lepA B. subtilis* at optimal (37°C) and low (24°C) temperatures. Disruptions of *bipA* and *lepA* genes were performed in Δ*efp* (strains BCHT175, BTHT17 and BTHT22 wt, Δ*bipA* and Δ*lepA* background, respectively), Δ*yfmR* (strains BCHT170, BTHT18 and BTHT23), Δy*diF* (strains BCHT174, BTHT19 and BTHT24), Δ*ykpA* (strains BCHT170, BTHT20 and BTHT25) and Δ*yfmM* (strains BCHT171, BTHT21 and BTHT26) *B. subtilis*. Bacteria were grown on solid LB medium at either 37°C (left panel) and 24°C (right panel) for 36 h. Wild-type *B. subtilis* as well as the isogenic Δ*bipA* (strain BCHT175) and Δ*lepA* (strain BCHT176) mutants were streaked as controls.

In our previous study of *E. coli* Uup, we have observed a modest genetic interaction between *uup* and *bipA*, with the simultaneous deletion of the two genes exacerbating cold sensitivity phenotype of the Δ*bipA* strain (33). We wondered is the same holds for *B. subtilis* YfmR/Uup. To test this, we have systematically disrupted the *abcf* genes in wild-type, Δ*bipA* and Δ*lepA* strains; the latter served as a specificity control. Since YfmR/Uup appears to have functional similarities to EF-P, we have also disrupted *epf* in all of the three genetic backgrounds. The strains were grown on solid LB either at optimal (37°C) and low (24°C) temperatures (Figure 8). In contrast to *E. coli*, we see no synthetic phenotype in Δ*bipA* Δ*yfmR* double-deletion strain. However, the Δ*bipA* Δ*epf* strain has a strong phenotype. At 37°C, while there is no clear growth defenct, the colonies are slightly translucent; at 24°C, the synthetic growth defect is clearly evident. Given the functional analogues between EF-P and YfmR/Uup, the phenotype is analogous to that we have earlier observed for Δ*uup* Δ*epf E. coli* (33).

## Discussion

The exact molecular functions of housekeeping ABCFs have been ‘a riddle wrapped in a mystery inside an enigma’ of bacterial protein synthesis for a decade. Housekeeping ABCFs are expected to resolve ribosomal stalls—but what kind of stalls? Several recent reports have provided important clues regarding the possible biological functions of both *E. coli* (43,64–66) and *B. subtilis* (67) factors. Hong and colleagues have reported that dCas9 knock-down of *yfmR* in Δ*efp B. subtilis* results a synthetic growth defect, increased ribosomal stalling on a pentaproline motif as well as accumulation of free 50S subunits (67). Furthermore, the authors showed that *E. coli* Uup can functionally replace YfmR/Uup in *B. subtilis*. Based on Tn-Seq results, Hong and colleagues concluded that simultaneous deletion of *yfmR* and *efp* could be synthetically lethal. However, by constructing a Δ*efp* Δ*yfmR B. subtilis* strain, here we show that the double-deletion strain is actually viable, although it does exhibit a serve growth defect. An elegant study by Chadani *et al.* has demonstrated that in a reconstituted PURE*flex* protein synthesis system (i) *E. coli* YbiT and EttA can suppress premature termination on negatively charged polyacidic motifs and (ii) *E. coli* Uup can alleviate ribosome stalling on polyproline stretches and (iii) simultaneous loss of Uup and EF-P results in a growth defect in *E. coli* (66). Finally, in good agreement with Chadani *et al.*, Ousalem *et al.* have revealed the role of *E. coli* YbiT in alleviation of the ribosomal stalling on acidic residues (65). All of these insights are well-aligned with our *in vivo* results with *B. subtilis*.

Despite recent progress, our understanding of bacterial housekeeping ABCFs is still incomplete. The contrast between, on the one hand, the exceedingly strong and specific genetic interaction between *efp* and *yfmR* and, on the other hand, the rather modest effects in stalling reporter assays is stark. Even more intriguing is the ability of the EF-P variant lacking the 5-amino-pentanolylated residue K32 to suppress the growth defect of the *efp* Δ*yfmR* strain. While the K32A EF-P variant is inefficient in resolving ribosomal stalling on polyproline, it is clearly competent in assisting YfmR in its biological function. The established function of modified lysine is stabilization of the P-site tRNA CCA end to promote the transpeptidation (4). Importantly, in addition to making contacts with the CCA end, EF-P specifically recognizes the D-arm of tRNA^Pro^ (4,68). Therefore, even while the K32A variant is compromised in reaching deep into the PTC, it still can potentially recognize the P-site tRNA identity. As simultaneous ribosomal association of the two E-site-binders is impossible, it is possible that YfmR and EF-P sequentially act on as-yet-unidentified proline-containing stalling motifs, with EF-P first positioning the P-tRNA^Pro^ followed by YfmR-mediated resolution of the stall. Furthermore, recent Ribo-Seq experiments have shown that overexpression of EF-P and its paralogue EfpL causes specific ribosomal stalling (9). Therefore, it could be that the function of EF-P is not to promote translation elongation, but to slow it down, thus presenting a relevant ribosomal substrate for YfmR. Finally, it is possible that the strong generic interaction between *efp* and *yfmR* is not due to the two factors working together in translation elongation at all. The eukaryotic EF-P orthologue, eIF5A, has been shown to play a key role in ribosome-associated quality control (RQC) (69), where the factor catalyses an elongation-like process on the large ribosomal subunit. Therefore, it is possible that there exists a yet-to-be discovered function of EF-P that does not require the modified K32 residue and is carried out in cooperation with YfmR.

In the absence a ‘smoking gun’, our highly reductionist reporter approach is incapable of identifying stalling motifs that require the assistance of ABCFs. Therefore, it is essential to apply global approaches such as ribosome profiling (70) or 5PSeq (71) to uncover the physiologically-relevant targets of *B. subtilis* housekeeping ABCF ATPases. Given the functional overlap between YfmR, YkpA and YfmM, the expression of individual ABCFs in Δ*4abcf B. subtilis* could be used to detect the subtle effects that would be otherwise masked in strains lacking only one of the ABCF factors. Once the native targets of YfmR and YkpA/YbiT are established, structure-functional studies will reveal the molecular mechanism of stall resolution by the ABCFs. Capitalising on the molecular insights into the mechanism of EF-P-mediated stimulation of PTC activity, modulation of EF-P concentration has been adapted as a strategy for improved efficiency of incorporation of non-canonical amino acids (72,73). It is possible that housekeeping ABCF ATPases could be useful for similar protein engineering applications in the future.

## Supplementary Material

gkae556_Supplemental_Files

## Data Availability

The data presented in this study are presented in the article itself as well as online Supplementary material.
