# Supplementary material for: Resolution of ribosomal stalling by EF-P and ABCF ATPases YfmR and YkpA/YbiT
Source: Nucleic Acids Res. 2024 Jun 29;52(16):9854–66. doi: 10.1093/nar/gkae556 (PMC11381351; doi:10.1093/nar/gkae556)
Supplement: gkae556_Supplemental_Files [file gkae556_supplemental_files.zip › Takada_SI.pdf]

SUPPLEMENTARY ONLINE MATERIALS

for

Resolution of ribosomal stalling by EF-P and ABCF ATPases YfmR and YkpA/YbiT

Hiraku Takada[1,2,3]*, Keigo Fujiwara[1], Gemma C. Atkinson[2,3], Chiba Shinobu[1], Vasili Hauryliuk[3,4,5,]*

[1] Faculty of Life Sciences and Institute for Protein Dynamics, Kyoto Sangyo University, Kamigamo, Motoyama, Kita-ku, Kyoto 603-8555, Japan

[2] Department of Biotechnology, Toyama Prefectural University, 5180 Kurokawa, Imizu, Toyama 939-0398, Japan

[3] Department of Experimental Medical Science, Lund University, 221 00 Lund, Sweden

[3] Virus Centre, Lund University, Lund, Sweden

[4] University of Tartu, Institute of Technology, 50411 Tartu, Estonia

[5] Science for Life Laboratory, Lund, Sweden

* to whom correspondence should be addressed:

Huraku Takada: hirakut58@pu-toyama.ac.jp

Vasili Hauryliuk, vasili.hauryliuk@med.lu.se

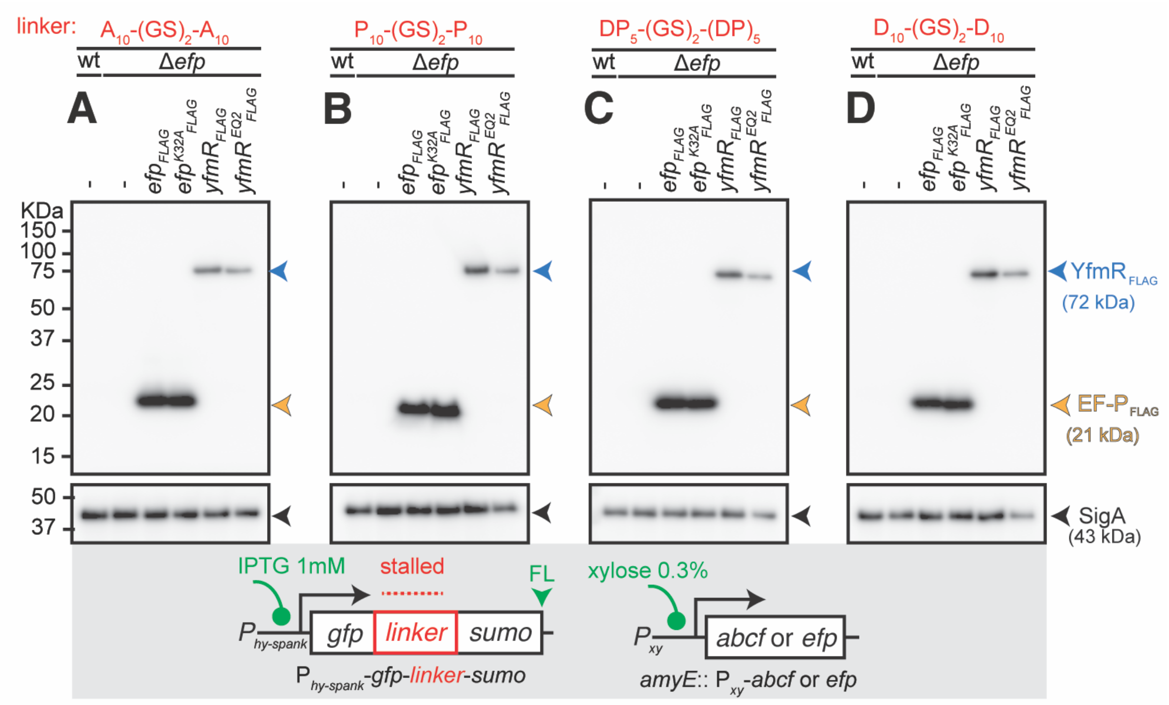

Supplementary Figure S1, related to Figure 5. Expression levels of wild-type and EQ_2 YfmR as well as wild-type and K32A EF-P assessed through anti-FLAG immunoblotting.

GFP-A_{10}-$(GS)_2$-A_{10}-SUMO (pCHT54) (**A**), GFP-P_{10}-$(GS)_2$-P_{10}-SUMO (pCHT55) (**B**), GFP-$(DP)_5$-$(GS)_2$-$(DP)_5$-SUMO (pCHT12) (**C**) and GFP-D_{10}-$(GS)_2$-D_{10}-SUMO (pCHT15) (**D**) reporters were expressed in wild-type, Δ*efp* (strain BCHT214) as well as in Δ*efp B. subtilis* expressing either efp_{FLAG} (strain BCHT1367), $efp^{K32A}{}_{FLAG}$ (strain BCHT1368), $yfmR_{FLAG}$ (strain BCHT1369) or $yfmR^{EQ2}{}_{FLAG}$ (strain BCHT1370) under the control of xylose promoter and both EF-P and YfmR were detected with anti-FLAG antibodies.

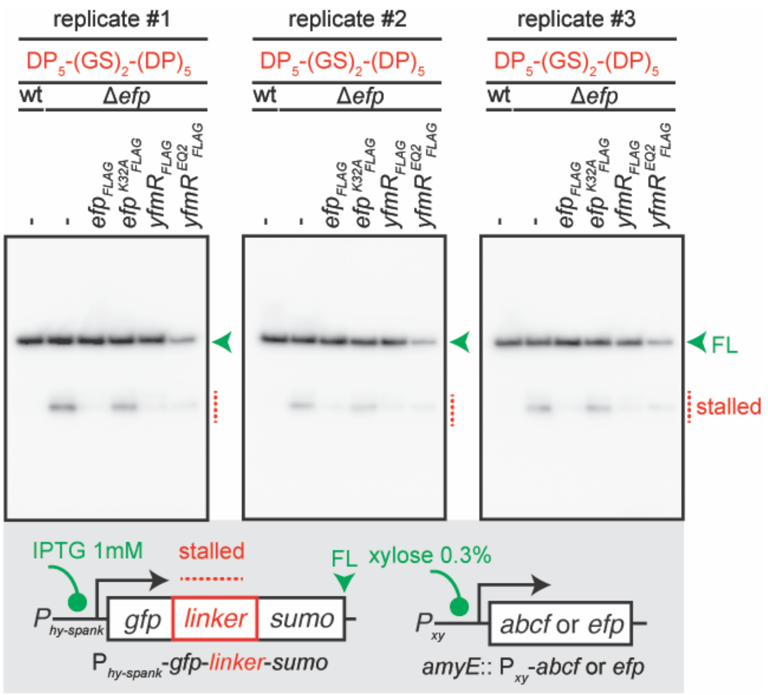

Supplementary Figure S2, related to Figure 5. Overexpression of YfmR/Uup ABCF alleviates the ribosomal stalling on Asp-Pro motifs: tree individual biological replicates.

Effects of EF-P and YfmR overexpression on ribosomal stalling on mixed Asp-Pro stalling motifs. The GFP-D_{10}-$(GS)_2$-D_{10}-SUMO (pCHT15) reporter was expressed in wild-type, Δ*efp* (strain BCHT214) as well as in Δ*efp* *B. subtilis* expressing either efp_{FLAG} (strain BCHT1367), $efp^{K32A}{}_{FLAG}$ (strain BCHT1368), $yfmR_{FLAG}$ (strain BCHT1369) or $yfmR^{EQ2}{}_{FLAG}$ (strain BCHT1370) under the control of xylose promoter and detected with anti-GFP antibodies. The full-length product is indicated with a green arrowhead and the stalled product is indicated with a red dotted line.

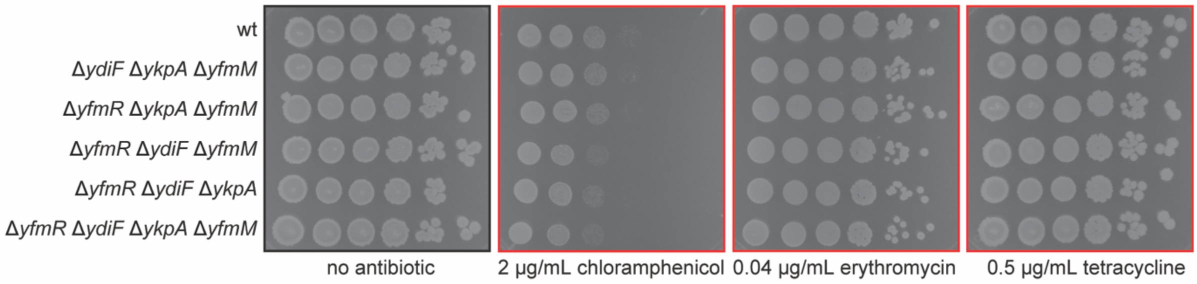

Supplementary Figure S3, related to Figure 7. Phenotypic characterisation of *B. subtilis* strains carrying multiple disruptions of ABCF genes.

Serial 10-fold dilutions of overnight LB cultures of wild-type, Δ*ydiF*Δ*ykpA*Δ*yfmM* (strain BCHT1388), Δ*yfmR*Δ*ykpA*Δ*yfmM* (strain BCHT1387), Δ*yfmR*Δ*ydiF*Δ*yfmM* (strain BCHT1386), Δ*yfmR*Δ*ydiF*Δ*ykpA* (strain BCHT1385) and Δ*yfmR*Δ*ydiF*Δ*ykpA*Δ*yfmM* (the Δ*4abcf* strain BCHT1389) strains were spotted onto either LB agar plates or LB plates supplemented with sub-MIC concentrations of translation-targeting antibiotics. The plates were sored after an 18-hour incubation at 37 °C.

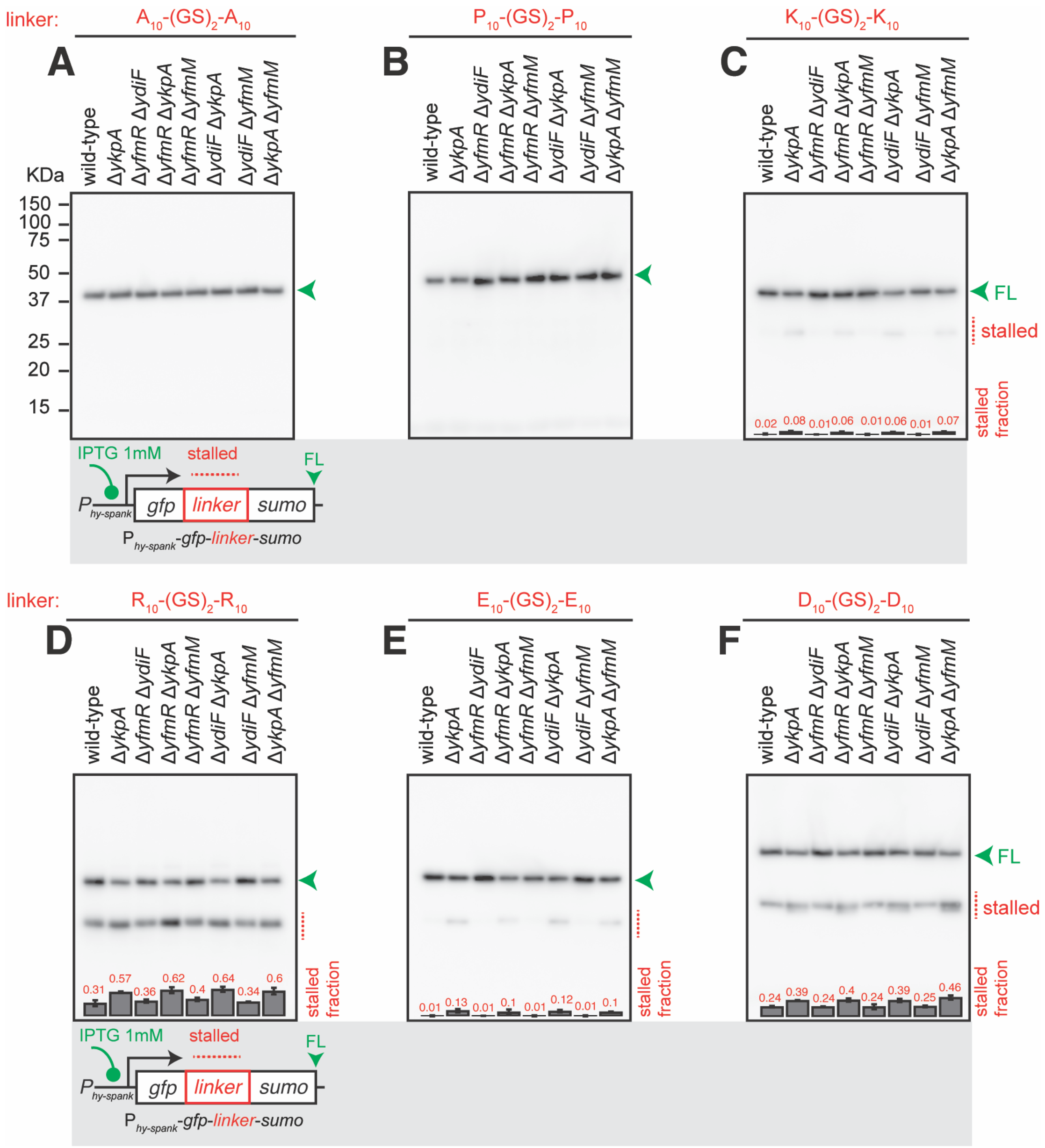

Supplementary Figure S4, related to Figure 7. Effects of combinatorial disruptions of ABCF genes on ribosomal stalling.

Effects of combinatorial ABCF gene disruption on ribosomal stalling on polyproline, Asp-Pro as well as negatively and positively charged homopolymeric motifs. GFP-A_{10}-$(GS)_2$-A_{10}-SUMO (pCHT54) (**A**), GFP-P_{10}-$(GS)_2$-P_{10}-SUMO (pCHT55) (**B**), GFP-K_{10}-$(GS)_2$-K_{10}-SUMO (pCHT13) (**C**), GFP-R_{10}-$(GS)_2$-R_{10}-SUMO (pCHT56) (**D**), GFP-E_{10}-$(GS)_2$-E_{10}-SUMO (pCHT11) (**E**) and GFP-D_{10}-$(GS)_2$-D_{10}-SUMO (pCHT15) (**F**) reporters were expressed in wild-type, Δ*ykpA* (strain BCHT215), Δ*ykpA* (strain BCHT215), Δ*yfmR*Δ*ydiF* (strain BCHT1379), Δ*yfmR*Δ*ykpA* (strain BCHT1380), Δ*yfmR*Δ*yfmM* (strain BCHT1381), Δ*ydiF*Δ*ykpA* (strain BCHT1382), Δ*ydiF*Δ*yfmM* (strain BCHT1383), Δ*ykpA*Δ*yfmM* (strain BCHT1384). The full-length product is indicated with a green arrowhead and the stalled product is indicated with a red dotted line. All reporters were detected with anti-GFP antibodies. Fraction of the stalled (short) product was quantified from three independent biological replicates and shown as mean ± standard deviation.
